# On the feasibility of malaria hypothesis

**DOI:** 10.1038/s41598-024-56515-2

**Published:** 2024-03-09

**Authors:** Farrokh Habibzadeh

**Affiliations:** Global Virus Network (GVN), Middle East Region, Shiraz, Iran

**Keywords:** Population genetics, Malaria, Sickle cell, Balanced polymorphism, Simulation, Population genetics, Evolutionary genetics

## Abstract

In 1954, Allison proposed that hemoglobin S (HbS) gene causes protection against fatal malaria. This would explain the high HbS gene frequency observed in certain regions hyperendemic for malaria, so-called “malaria hypothesis”. This in silico study was conducted to examine the feasibility of the hypothesis under more realistic initial conditions, where a mutant gene with heterozygous advantage against malaria (e.g., HbS) was introduced in a group of Neolithic hunter-gatherers who decided to start agriculture nearby water where malaria killed a proportion of population. The tribe population size, number of children born to each woman in each generation, mortality from malaria and sickle cell disease, the protection factor provided by the gene carriers against malaria, the probability of mating between the members of the parent and offspring populations, population growth, and increased fertility in women heterozygous for HbS, were also considered. For effectively confer protection against malaria within the shortest possible period, the mutation needs to be happened in a small population. For a large population, the process would take around 100 generations (~ 2500 years) or more to provide an effective protection. Even then, the probability that the new gene could survive and propagate to future generations is about 35%. Conventional population genetics equations with differential or difference equations, give totally incorrect estimates of the gene frequency in small populations; discrete mathematics should be used, instead. After introduction of the advantageous mutation, the gene frequency increased until a steady state value. This value is far less than the gene frequency reported in certain tribes of Africa. It seems that the malaria hypothesis, per se, could not explain such a high observed gene frequency, unless HbS is associated with lower mortality from other causes too.

## Introduction

Thalassemia and sickle-cell anemia are examples of autosomal recessive monogenic hereditary hemoglobinopathies with certain distributions across the globe. The very high gene frequencies of carriers for thalassemia and sickle-cell anemia in some regions, found in population surveys for these disorders in the post-World War II era, puzzled population geneticists^1^. In some tribes in Africa, the sickle cell trait (heterozygous form of the disease) is present in as much as 40% of the population^2,3^; 4% have sickle cell disease (homozygous form of the disease), which gives a gene frequency (*f*_*gene*_) of 24%^4–6^. With no treatment, most of children homozygous for this gene die of the associated complications and cannot survive to the reproductive age; this, in turn, results in more than 10% loss of the defected genes after each generation. Nonetheless, the *f*_*gene*_ has remained high without any sign of declining for several decades^5,7^.

In 1946, in a study on patients at a regional hospital in Zambia (previously, Northern Rhodesia), Beet recorded lower rates of malarial infection among carriers of the sickle cell trait than amongst non-sicklers^8^. Two years later, Haldane proposed an explanation for the high frequencies of thalassemia around the shores of the Mediterranean Sea, where malaria had long been endemic^4,9^. He asserted that “the corpuscles of the anemic heterozygotes are smaller than normal and more resistant to hypotonic solutions. It is at least conceivable that they are also more resistant to attacks by sporozoa which causes malaria, a disease prevalent in Italy, Sicily and Greece, where the gene is frequent”^9^. In 1954, Allison proposed that hemoglobin S (HbS) gene also causes protection against fatal malaria^2,10,11^. By the end of 1960’s, it was generally accepted that the high HbS gene frequency reflects heterozygote advantage against fatal malaria caused by the parasite *Plasmodium falciparum*; this relationship became the prototype of a phenomenon so-called “balanced polymorphism” in man — *f*_*gene*_ for the advantageous heterozygous state increases until its incidence is balanced by the loss of homozygotes from the population. Currently, this hypothesis, which is known as “malaria hypothesis”, has gained enough acceptability that many authorities have stated that “we now have enough knowledge to say with some confidence that what was dubbed the ‘malaria hypothesis’ for so many years is no longer a hypothesis”^1^.

To understand various aspects of this hypothesis, many researchers have used population genetics equations to compute the *f*_*gene*_ at different times. Most of the equations are easy to follow and can be found in basic population genetics texts^12^. For instance, if HbS gene frequency has a value of *p*, assuming a binomial distribution, the frequencies of homozygous (*f*_*homo*_), heterozygous (*f*_*hetero*_) and normal (*f*_*normal*_) individuals in the population at the zygotic stage are *p*^2^, 2 *p* (1 – *p*), and (1 – *p*)^2^ for alleles of *SS*, *AS*, and *AA* (*S* is the diseased and *A* the non-diseased allele), respectively. If the fitness (*W* ) of these genotypes is designated as *W*_*SS*_, *W*_*AS*_, and *W*_*AA*_, respectively, the *f*_*gene*_ at the *i*th generation, *p*_*i*_, can be calculated from *f*_*gene*_ at the previous generation, *p*_*i–*1_, according to the following difference equation^12^:

1 $$p_{i} = \frac{{p_{i - 1}^{2} W_{SS} + p_{i - 1} \left( {1 - p_{i - 1} } \right)W_{AS} }}{{p_{i - 1}^{2} W_{SS} + 2\,p_{i - 1} \left( {1 - p_{i - 1} } \right)W_{AS} + \left( {1 - p_{i - 1} } \right)^{2} W_{AA} }}$$

The change in *f*_*gene*_ becomes zero at a gene frequency of 0 (no diseased gene in the population), 1 (all the alleles in the population contain the defected gene), or when the *f*_*gene*_ reaches the equilibrium value of:

2 $$p_{equilibrium} = \frac{{W_{AA} - W_{AS} }}{{W_{AA} - 2 \, W_{AS} + W_{SS} }}$$

While Eqs. (1) and (2) were obtained assuming an infinite population size, Eq. (2) works for all population sizes.

Some researchers have used computer simulation to explore more complicated situations^13–16^. Some simulations took into account that the population has a finite size^17,18^; in many studies, however, the authors overlooked several important variables. For instance, they have not considered that the studied population is not infinite, that the parent and offspring population might have degrees of overlap (some people in the parent population may mate with members of the offspring population), that not all of those with sickle cell anemia die of the disease (some may survive and give birth to children), and that the population may grow in size over generations, to mention just a few limitations of such reports. The current in silico study was conducted to examine the feasibility of “malaria hypothesis” under different initial conditions for the above-mentioned variables.

## Results

**Figure 1:**
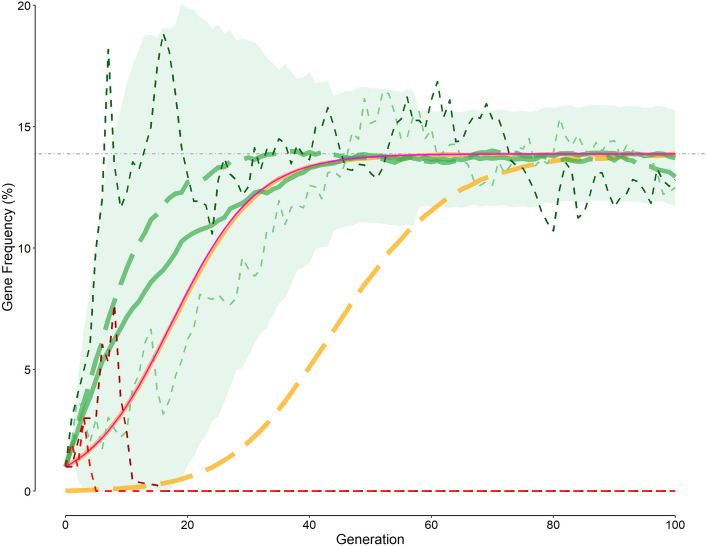
Variation of the gene frequency over generations. Green and red dashed curves are examples of four arbitrarily chosen rounds of the simulation—green dashed curves for those succeeded; red dashed curves, those aborted. The green solid curve is the result of the simulation with the default initial values; (Table 1, scenario 6: 25 couples, a new mutation introduced); the light green region represents the 95% confidence interval for the curve (scenario 6); green long-dashed curve, simulation results of scenario 2 (25 couples, a new mutation introduced, no growth, no overlap); orange solid curve, simulation results of scenario 1 (2500 couples with an initial gene frequency of 1%); magenta solid curve, the theoretically calculated frequency (Eq. 1); orange long-dashed curve, simulation results of scenario 3 (2500 couples with a new mutation introduced). The horizontal gray dot-dashed line represents the equilibrium gene frequency (Eq. 2).

Temporal changes of *f*_*gene*_ over generations under eight studied scenarios (Table 1) are shown in Fig. 1. In all the studied scenarios, *f*_*gene*_ reached an equilibrium value, which was consistent with the predicted value (Eq. 2). However, the way *f*_*gene*_ changes over time was different from scenario to scenario. The fitness of *SS*, *AS*, and *AA* genotypes was calculated as follows:

3 $$\begin{gathered} W_{AA} = 1 - M_{m} \hfill \\ W_{AS} = 1 - \frac{{M_{m} }}{{P_{hetero} }} \hfill \\ W_{SS} = 1 - \left[ {M_{SS} + \left( {1 - M_{SS} } \right)\frac{{M_{m} }}{{P_{homo} }}} \right] \hfill \\ \end{gathered}$$

where *M*_*m*_ represents the probability a person with *AA* genotype (normal) dies of malaria before the reproduction age, assumed to be 15% (the default value); *M*_*SS*_, the probability a person with *SS* genotype (homozygote) dies of sickle cell disease before the reproductive age, assumed to be 85%; and *P*_*hetero*_ and *P*_*homo*_, the protection conferred by *AS* and *SS* genotypes against malaria, respectively, assumed to be 10 for both genotypes.

**Table 1:** The initial values for the simulation in various scenarios studied.

Scenario	Initial couples	Life style*	*M*_*m*_ (%)	Gene abortion probability (95% CI^†^) (%)
1^‡^	2500	–	15	0
2	25	–	15	86.8 (86.5–87.1)
3	2500	–	15	69.5 (68.0–71.0)
4	25	5	5	79.1 (78.7–79.5)
5	25	5	10	71.9 (71.4–72.3)
6^§^	25	5	15	64.1 (63.6–64.6)
7	25	5	20	59.3 (58.8–59.8)
8	25	5	25	54.1 (53.6–54.6)

For all scenarios a malaria mortality rate (*M*_*m*_) of 15%, a protection of 10 times for both heterozygous and homozygous individuals, and a mortality rate of 85% for those with sickle cell disease (*SS* homozygotes) were assumed. For the first three scenarios no overlap between the parent and offspring populations, no population growth, and no change in life style (switching from a hunter-gatherer to a farmer) were assumed. For scenarios 4–8, It was assumed that the population will begin to grow and the life style (third column) will be changed from the fifth generation onward. The population grow will continue after the population reaches a maximum of 1000 couples. A fixed population overlap of 5% from the first generation onward was also assumed.

*Generation when life style begins.

^†^Confidence interval.

^‡^An initial gene frequency of 1% was assumed.

^§^Default initial values.

### The gene frequency

#### Predictions from classic population genetics equations

The temporal change of *f*_*gene*_ computed from Eq. (1), assuming an initial *f*_*gene*_ of 1% is presented in Fig. 1 (magenta solid curve). The frequency increased smoothly and reached the equilibrium value (Eq. 2).

#### Scenario 1

Starting with an initial *f*_*gene*_ of 1% in a population with 2500 couples at reproductive age, no overlap between the parent and offspring populations, and no population growth (Table 1), the *f*_*gene*_ increased over generations (Fig. 1, orange solid curve underneath the magenta solid curve). The results were exactly the same as those predicted from Eq. (1); after almost 50 generations (more than 1200 years), the frequency reached the equilibrium (Fig. 1, horizontal gray dot-dashed line).

#### Scenario 2

All the initial values were the same as those for scenario 1, except that the study population was consisted of 25 (instead of 2500) couples at reproductive age. The initial *f*_*gene*_ of 1% was equivalent to the introduction of a single mutation in the population. The temporal variation of *f*_*gene*_ significantly differed from that predicted from Eq. (1) and scenario 1 (initiating with 2500 couples); the frequency increased rapidly over generations (Fig. 1, green long-dashed curve) so that after almost 30 generations (~ 700 years), it reached the equilibrium (Fig. 1, horizontal gray dot-dashed line).

#### Scenario 3

The initial parameters were just similar to those for scenario 1 except that the initial *f*_*gene*_ was not assumed to be 1%; it was assumed that like scenario 2, a single mutation happened, which given a population size of 5000 persons (10,000 gene alleles) translated into an initial *f*_*gene*_ of 0.01% (Table 1). The *f*_*gene*_ increased over generations (Fig. 1, orange long-dashed curve), but at a much lower pace compared with scenario 1; after almost 90 generations (~ 2200 years), the frequency reached the equilibrium (Fig. 1, horizontal gray dot-dashed line).

#### Scenario 4–8

In these scenarios 25 couples, 5% overlap between the parent and offspring populations, and population growth were assumed. The only difference between these scenarios was in the mortality rate for malaria assumed for each scenario, which was 5%, 10%, 15%, 20%, and 25% for scenarios 4 to 8, respectively. Here again, the *f*_*gene*_ increased over generations; for example, the green solid curve in Fig. 1 is the temporal variation in *f*_*gene*_ assuming a malaria mortality rate of 15% (Table 1, scenario 6). In 58 of 10,000 rounds of simulation of scenario 6 (the default values), the *f*_*gene*_ sometimes became 24% or more. In another words, under scenario 6, the probability that *f*_*gene*_ reached 24% or more was 0.0058, hence (p = 0.0058).

### Elimination of the gene from population

For all scenarios studied, but scenario 1, there was a chance of failure. Not all rounds of the simulation led to a steady increase in the *f*_*gene*_; sometimes, those carrying the newly-introduced gene (either those homozygous or heterozygous for the gene) were eliminated from the population for any reasons before passing their gene to their offspring (in this simulation, dying of sickle cell disease complications or of malaria; in real life, they may die of other causes too). The *f*_*gene*_ dropped to zero, like that the gene was “aborted” (Fig. 1, red dashed curves). Sometimes, the gene could propagate to next generations and ultimately reach the equilibrium (Fig. 1, green dashed curves).

**Figure 2:**
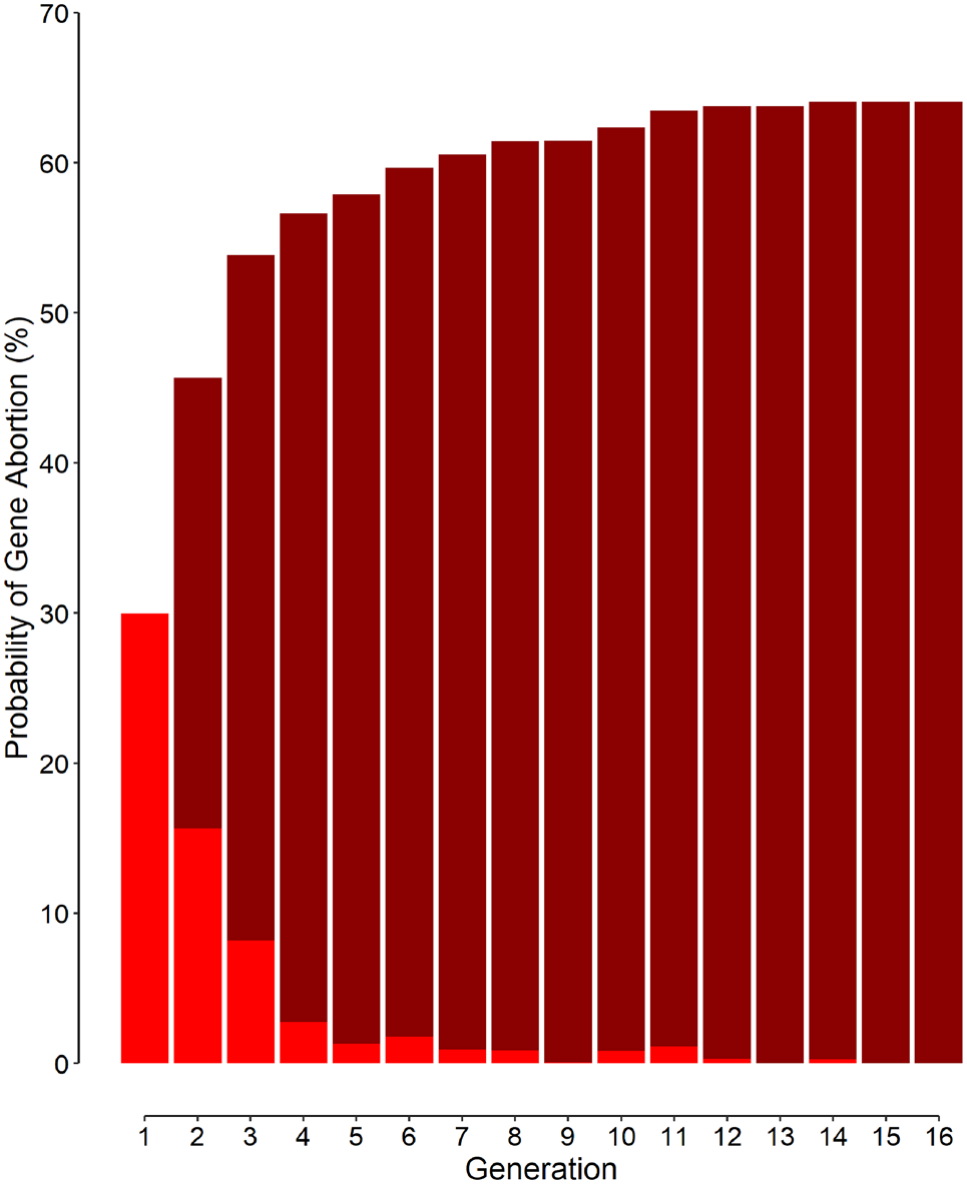
The probability of gene abortion given the scenario 6 (Table 1). Light red bars represent the probability for each generation; dark red bars, the cumulative probability.

**Figure 3:**
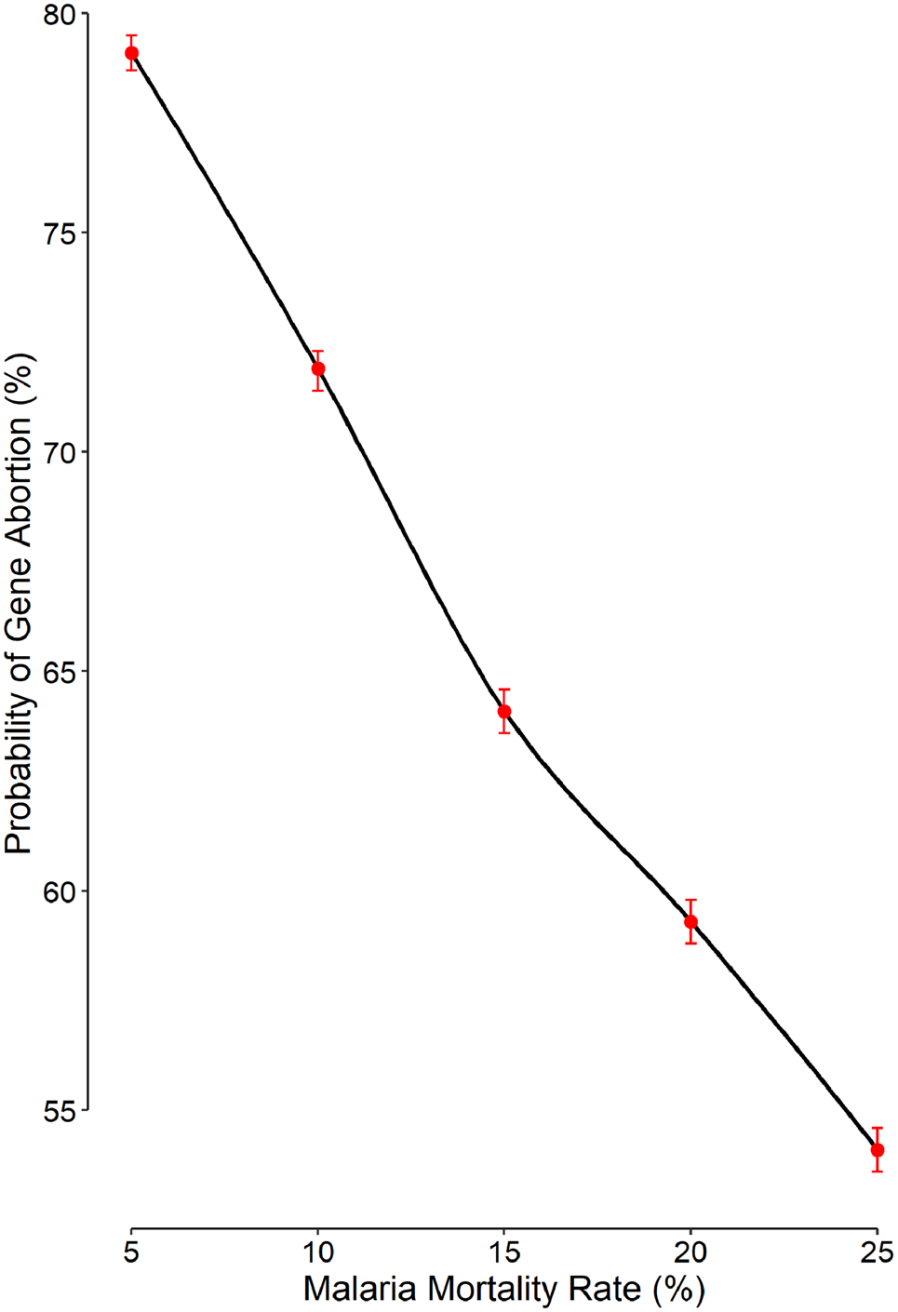
Cumulative probability of gene abortion for various malaria mortality rates. The error bars represent the 95% confidence interval.

The cumulative probability of gene abortion in scenario 6 was 64.1% (Table 1). Most of the abortions happened early (Fig. 2) — 30.0% (95% confidence interval, 29.1% to 30.9%) in the first generation; 15.7% (15.0% to 16.4%), second generation; and 8.2% (7.7% to 8.7%), third generation (Fig. 2). In scenario 6, abortion could happen even in the 16th generation. According to simulation results, the cumulative probability of the gene abortion linearly decreases with increasing malaria mortality (Fig. 3).

**Figure 4:**
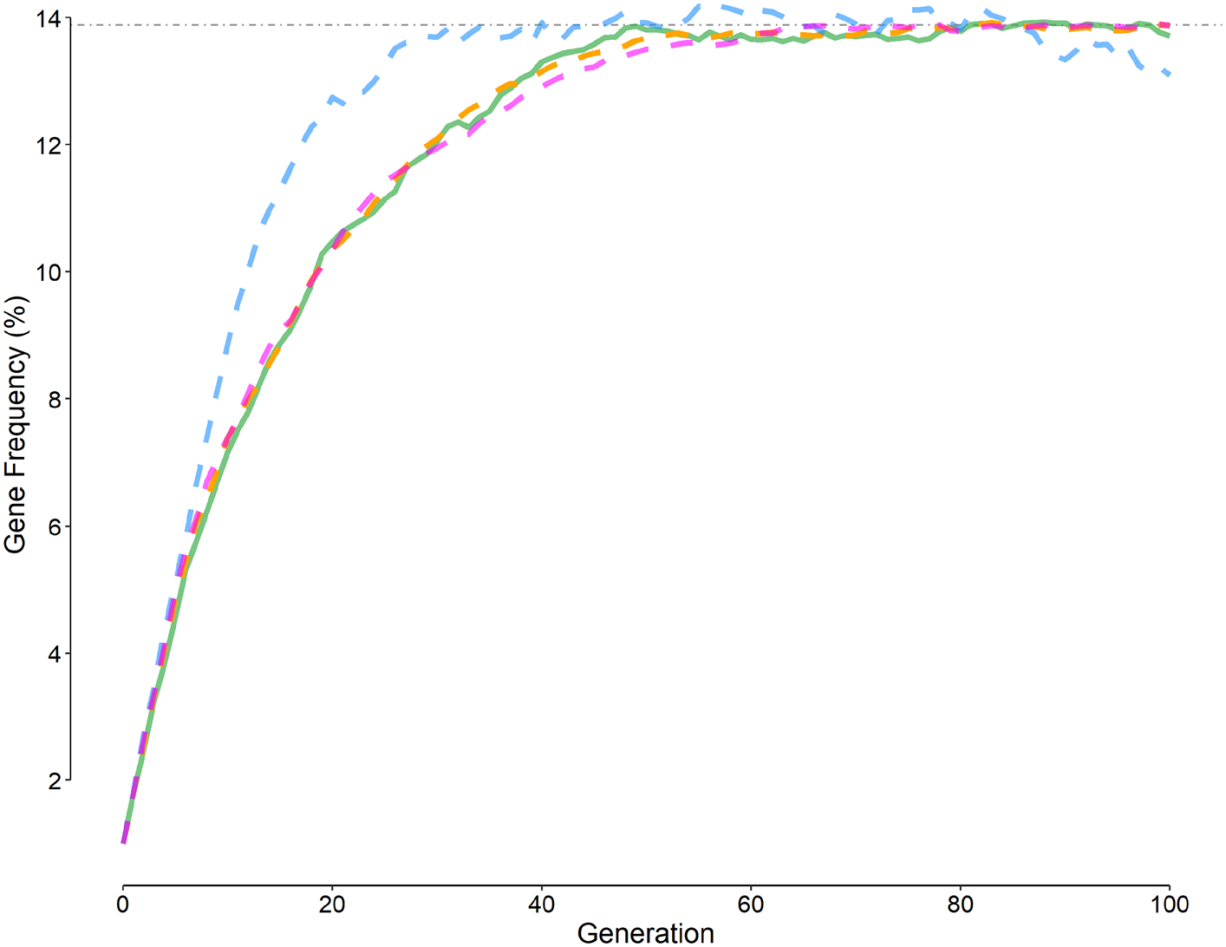
Temporal variation in the gene frequency under several initial conditions (Table 2). The green solid curve is the frequency given by scenario 6 (Table 1, default initial values); magenta dashed curve, scenario 6 and assuming that heterozygous individuals (*AS* genotype) had a 10% higher fertility rate compared with normal people; orange dashed curve, scenario 6 without overlap between the parent and offspring populations; and blue dashed curve, scenario 6 but no population growth. The horizontal gray dot-dashed line represents the equilibrium gene frequency (Eq. 2).

Assuming that there is no overlap between the parent and offspring population or that those with *AS* genotype have a higher fertility rate (e.g., 10% more) than normal people did not change the results of simulation (Fig. 4). However, the *f*_*gene*_ had a steeper rise when it was assumed that the population size did not grow (Fig. 4, blue solid curve).

## Discussion

The classic population genetics models mainly rely on differential and difference equations. The former approach presumes that both *f*_*gene*_ and time (generation) are continuous variables. The latter assumes that while time is a discrete variable, *f*_*gene*_ is continuous. Although the methods work properly when the population size is large (e.g., Table 1, scenario 1). Assuming an initial gene frequency of 1% in a population with 2500 couple at reproductive age, the predicted *f*_*gene*_ at each generation computed from the simulation (Fig. 1, orange solid curve) was the same as the value derived from classic population genetics equations (Eq. (1); Fig. 1, magenta solid curve). The frequency obtained from the simulation for exactly the same initial values but a population size of 25 couples at reproductive age (Table 1, scenario 2; Fig. 1, green long-dashed curve) was nevertheless very different from those derived based on classic population genetics equations, such as Eq. (1) (Fig. 1, magenta solid curve). The observed difference between the results of the two scenarios is due to the assumption of continuity of *f*_*gene*_. While for a large population (e.g., scenario 1 with initial 2500 couples) the frequency may be assumed to be continuous, for a small population (e.g., scenario 2 with initial 25 couples), this assumption is no longer valid. For instance, in a population of 25 couples (50 people with 100 alleles), *f*_*gene*_ can only assume multipliers of 1/100 (i.e., 1%, 2%, … , 99%, 100%); it can never be 1.3% or 2.7%, for example. Working with such quantal discrete values requires discrete mathematics, not differential or difference equations such as Eq. (1) that assume continuity of *f*_*gene*_. The simulation program used in this study takes this important issue into account (Table 3). This is why the results of the simulation and classic population genetics equations are similar for a large population (where the stepwise change in *f*_*gene*_ can be ignored and *f*_*gene*_ can be treated as a continuous variable).

A new mutation introduced is not necessarily going to spread rapidly in a population, unless it is associated with a highly selective advantage. Even so, the new gene is subject to considerable drift in the first few generations; there are only one or a few individuals carrying the new mutated gene. During this early period, it is not unlikely that the gene being eliminated from the population, even if it is very advantageous^19^. The *f*_*gene*_ in a population with 25 couples (100 gene alleles) cannot be lower than 1%; the lower value the *f*_*gene*_ can assume is zero; unlike the results of Eq. (1), the frequency cannot be 0.7%, 0.01%, or any value between zero and 1%. This is why when a mutation happens in a small population (Table 1, scenarios 2 and 4–8 with initial 25 couples), the discrete* f*_*gene*_ can easily drop to zero and vanish (Fig. 1, red dashed curves), for example by dying of malaria (although at a lower rate compared with normal people). The probability that the introduced mutant gene becomes aborted is particularly high during the first generations (Fig. 2). For scenario 6, the probability of gene abortion is 30% in the first generation; the abortion may happen, although with lower probabilities, even 16 generations after the introduction of the mutation (Fig. 2). The cumulative probability of gene abortion is 64.1% (Table 2).

**Table 2:** The value of some of the parameters after 100 generations (at equilibrium).

Parameter	Default values, scenario 6	All variables set as default (scenario 6), but …		
		No overlap	No population growth	Increased fertility by 10%
Mean (SD) *f*_*gene*_ (%)	13.7 (1.3)	13.9 (1.2)	13.1 (7.4)	13.9 (1.2)
Mean (SD) *f*_*hetero*_ (%)	23.7 (1.9)	23.9 (1.8)	22.0 (11.2)	23.9 (1.8)
Mean (SD) total mortality (%)	12.4 (0.8)	13.1 (0.8)	13.0 (4.9)	12.5 (0.7)
Probability of gene abortion (95% confidence interval) (%)	64.1 (63.6–64.6)	63.0 (62.5–63.5)	88.9 (88.5–89.2)	66.9 (66.4–67.4)

The default simulation parameters included a population size of 25 couples (50 individuals) at the reproductive age, a malaria mortality rate (*M*_*m*_) of 15%, a protection of 10 times for both heterozygous and homozygous individuals, a mortality rate of 85% for those with sickle cell disease (*SS* genotype), a mean of 4 (hunter-gatherer phase) and 5 (farmer phase) children in each generation, 5% overlap between parent and offspring generations, a growing population, and no increased fertility when one or both of the parents was heterozygous (*AS* genotype).

The probability that a mutant gene being aborted is not only considerable in small populations; it is high even in large populations. The probability of gene abortion in the first generation is also 30% for scenario 3 (with initial 2500 couples; Fig. 1, orange long-dashed curve); with a cumulative probability of 70%, the abortion may even occur as late as the 44th generation.

The cumulative probability of the gene abortion decreased almost linearly with increasing malaria mortality rate (Fig. 3). For low mortality rates, the protection conferred by the advantageous gene is not significant; the gene frequency would therefore decline, even to zero (gene abortion). On the other hand, for higher mortality rates, presence of the advantageous gene would become much important; most normal people die of malaria and the gene frequency will typically rise quickly. A higher gene frequency decreases the risk of gene abortion.

Occurrence of a mutation that confers protection against malaria is very rare. Even if it happens in a large population (e.g., scenario 3; Fig. 1, orange long-dashed curve), it takes almost 90 generations (~ 2200 years) that *f*_*gene*_ reaches the equilibrium and provides maximal protection against malaria. The time course is almost similar to that found in another in silico study which shows that *f*_*gene*_ would reach the equilibrium value of 11.7% after 45 to 70 generations, if a new mutation with a heterozygous advantage (*W*_*AS*_* / W*_*AA*_ of 1.152) occurs in a population with 500 couples^17^. The lower predicted time to reach to the equilibrium state compared to scenario 3 in our study (Fig. 1, orange long-dashed curve) is due to the difference in the population size. Although malaria hypothesis would work in a large population, to be effective and in a way, feasible, the protection to be conferred against malaria should occur in the shortest possible period. To provide the protection as soon as possible, the most feasible way is that the mutation occurs in a small tribe (e.g., scenario 6) rather than a large population.

Given the size of common populations in the world today and assuming a mutation rate of 10^–9^ per nucleotide, the same mutation may occur in more than one individual in every generation. A few thousand years ago, the human population was much smaller than it is now. At the end of the Paleolithic era, the population was much smaller. Under scenario 6 (Fig. 1, green solid curve), it took almost 45 generations (more than 1000 years) for *f*_*gene*_ to reach the equilibrium. On the other hand, although there was a considerable chance of elimination of the mutated gene, there was probable that the mutated gene rapidly spread in the population within just 4 or 5 generations (about 100 years) to a frequency to effectively protect people (Fig. 1, the dark green dashed curve).

Assuming that there was no overlap between the parent and offspring population or that those heterozygous for HbS gene had a higher fertility rate (e.g., 10% more) than normal people did not change the results of simulation (Fig. 4). However, the *f*_*gene*_ had a steeper rise when it was assumed that the population size did not grow (Fig. 4, blue dashed curve). Keeping the population size small resulted in higher gene frequencies after generations. The *f*_*gene*_ may decrease in a growing population.

The equilibrium *f*_*gene*_ value of 13.9% predicted from Eq. (2) was very near to that of simulation results (Fig. 1). This equilibrium frequency depends on the amount of advantage conferred by the mutant gene against malaria (*W*_*AS*_* / W*_*AA*_ of 1.159) for scenarios 1–3, and 6 (the default scenario), which was very close to the values obtained by other researchers^17,18,20^.

Under all scenarios studied, the *f*_*gene*_ was not constant after reaching the equilibrium state; its value fluctuated with time around the equilibrium frequency. This is indeed the well-known genetic drift observed in a population^12^. The magnitude of the variation around the equilibrium was random with a maximum variation of around 5%. Under scenario 6, the probability that *f*_*gene*_ reached 24% or more was 0.0058. Therefore, it can be inferred that it is very unlikely (p = 0.0058) that given the default initial values (scenario 6) the observed gene frequency reaches 24% or more. The observed *f*_*gene*_ of 24% recorded in some African tribes^4–6^, could thus not be explained by the malaria hypothesis unless the presence of the mutant gene could also confer protection against other disease conditions and decrease the mortality.

In a cohort study conducted in Africa, Aidoo et al., have shown that the *AS* genotype is associated with a lower all-cause mortality among 12–16 month-old children^21^. A recent study has also shown that malaria can confer non-specific protection against other infections, such as severe acute respiratory syndrome coronavirus 2 (SARS-CoV-2), probably through stimulation of innate immunity^22^. While the protection conferred by the mutant gene is associated with a higher *f*_*gene*_ equilibrium, the protection provided by malaria is expected to result in a decrease in the equilibrium frequency. The equilibrium state (presumably, where the all-cause mortality rate is a minimum) is then not only determined by the mortalities attributable to the sickle cell disease and malaria, but also depends on the fatalities caused by other disease conditions in the region, which could be altered by the non-specific protections conferred by *AS* genotype and malaria. These issues should be examined in detail in forthcoming studies.

In conclusion, the malaria hypothesis can happen in any population regardless of its size. However, to be feasible and for effectively confer protection against malaria within the shortest possible period, the advantageous mutation should most likely occur in a small population. In a large population, it takes a very long time the frequency of the new gene gets large enough to provide a sound protection. Even then, the likelihood that the gene propagates to next generations and confers protection is not high. In analyzing the trend of gene frequency in such setting, discrete mathematics should be used; differential and difference equations will give misleading results. Different fertility of those heterozygous for the gene and the extent of overlap between the parent and offspring populations do not significantly affect the temporal trend of the gene frequency and may not be considered in future simulations. To correctly compute the magnitude of protection conferred by *AS* or *SS* genotypes against malaria, the non-specific protections provided by the mutant gene and malaria against other infections prevalent in the region should also be taken into account.

## Methods

### Monte Carlo simulation

Monte Carlo simulation is a type of stochastic simulation that incorporates random variability into the model. Monte Carlo simulation differs from traditional simulation in that the model parameters are treated as stochastic or random variables, rather than as fixed values. Monte Carlo simulation repeatedly simulates the model, each time drawing a different random set of input values from a set of possible values to determine the resultant set of possible outcomes^23^.

#### Factors considered

To have a valid realistic simulation we should identify the important variables and estimate their effects on the process. To simulate the condition for testing the malaria hypothesis, let us assume that a group of Neolithic hunter-gatherers decided to start agriculture nearby water where malaria killed a proportion of population before the reproductive age. Assume that a mutant gene, with heterozygous advantage against the deadly malaria (say HbS) appeared in this population. Let assume that while this mutation in its homozygous form would kill most of the affected persons before the reproductive age, those heterozygous for the gene would be protected against the fatal malaria. Based on Mendelian inheritance rules, it is not difficult to figure out what would happen to this protective mutant gene over the next generations.

The variables considered in this simulation included the tribe population size, number of children born to each woman in each generation, population growth over generations, mortality from malaria (*M*_*m*_) and sickle cell disease (*M*_*SS*_), the protection factor conferred against malaria by the heterozygous (*P*_*hetero*_) and homozygous (*P*_*homo*_) states, the probable increase in fertility rate of women with *AS* genotype, and the presence of any overlaps between the parent and offspring populations (i.e., the percentage of the parent population which may mate with members of the offspring population).

##### Population size

In almost 8000 years ago, the Neolithic agrarian revolution has begun in the “Fertile Crescent,” southern Turkey. This movement was extended to western and Central Africa around 4000 to 5000 years ago^24^. In adopting an agricultural way of life, human population in sub-Saharan Africa changed from low-density and mobile hunter-gatherer life-style to communal living in settlements, expectedly, nearby water where malaria had long been waiting for them. The *Anopheles* vectors of human malaria evolved to adapt this situation. The work demands of such a tribal life, however, require a minimum number of adults in a village to provide for the dependents, share the work load, and cover the needs of families in case of adult accident or illness^25^. The size of hunter-gatherer groups of people was not high. For the current simulation, a default village population size of 150 persons was assumed. Given that almost one-third of the individuals in a population are of parenting age, the hypothetical village had an “effective population size” of 50 adults (25 men and 25 women) in reproductive age^19,25^.

##### Mean number of children born to each woman in each generation

Each couple of the hunter-gatherers gave birth to an average of five children — one about every 4 years^26^. With this spacing, parents can carry the youngest child, while the older children who can already walk follow the tribe at a reasonable pace. Then, children were breast-fed for a longer period which in turn lowered the probability of another pregnancy^27^. The mean number of five children born to each woman during her whole reproductive life kept the population size of the hunter-gatherers substantially stable since more than half of the children died early before the reproductive age^26^. Although, there is no exact value proposed for the average number of children born to each woman and the value is different from report to report for different tribes^25,26,28^, for this simulation, it was assumed that 10%, 15%, 50%, 15%, and 10% of couples in the simulated hunter-gatherer population gave birth to 2, 3, 4, 5, and 6 children, respectively. The percentages are indeed the values of the probability mass function for the distribution of the number of children each couple would have. This translates into a mean number of four children for each couple.

As agriculture expanded, food became abundant and farmers had no reason to limit the number of their children. They were settled and did not have to move from place to place^26^. The population size grew rapidly. On the other hand, with increasing population size, contagious diseases surfaced and caused more deaths^29^. Nonetheless, the net effect caused an increase in the mean number of children^26^. In this simulation, it was assumed that for the first five generations during which the hunter-gatherers learned effective agriculture, the mean number of children did not change and that the distribution of children was remained as it was described earlier. However, from the fifth generation onward, it was assumed that the mean number of children increased to five for each couple — 10%, 15%, 50%, 15%, and 10% of couples in the simulated farmer population gave birth to 3, 4, 5, 6, and 7 children, respectively.

##### Population growth

The hunter-gatherers had an almost stationary population size^19,25,26^. However, with abundance of food and increasing number of children, the population grew. The following logistic function was used to estimate the population size at each generation:

$$N_{t} = \frac{{1000N_{0} \, e^{0.15t} }}{{1000 + N_{0} (e^{0.15t} - 1)}}$$

where *N*_*t*_ designates number of couples in reproductive age, *N*_0_ number of couples in reproductive age at first generation (i.e., 25 couples), and *t* number of generations past after start of the growth — the fifth generation. The population was assumed to be stationary (i.e., 25 couples in reproductive age) during the first five generations, and grow thereafter until the time when the effective population size (those in reproductive age) for limited resources available reached a maximum of 1000 couples (i.e., total population of 6000 persons).

##### Mortality from malaria (*M*_*m*_)

In endemic areas with stable malaria, because of regular inoculations every person receives, a strong protective immunity against overt illness and risk of death from malaria is acquired usually by the age of 4 or 5 years. Therefore, most of the morbidities and mortalities occur before this age^30,31^. This is especially true in the presence of *P. falciparum.* Historically (and still today), such conditions have prevailed mainly in sub-Saharan Africa^24^.

Determination of malaria mortality rates in settled human populations living in endemic areas without access to drug treatment is very difficult and is usually done through three ways: (1) information collected from direct observation of deaths within a community; (2) data from malaria eradication and its effect on total mortality; and (3) calculation of the expected mortality rate that can be deduced from the gene frequency of alleles which cause protection against malaria^31^. Obviously, in the current study the third method could not be used, since it depends on the “malaria hypothesis”, itself, the validity of which is tested herein. Estimations of the mortality rate reported differently from study to study from 5 to 114 deaths per 10,﻿000 population per year^2,24,30–35^.

Considering that most of malaria mortalities occur before the age of five years, it was assumed that any newborn in the hypothetical simulation population ran a risk of 15% to die of malaria before the reproductive age.

##### Mortality from sickle cell disease (*M*_*SS*_)

In 1960, sickle cell disease was essentially treated as a disease of childhood. Its mortality was reported very high so that only few patients reach adult life^36,37^. Allison stated that only about 20% of children with sickle cell disease survived to reproductive age^5^. Some of the survivor males may also suffer from infertility^38^. For the current simulation, it was assumed that the likelihood that a person with sickle cell disease would die of the disease complications before the reproductive age was 85%.

##### Protection factor (*P*_*hetero*_ and *P*_*homo*_)

Children in West Africa who have *AS* genotype (sickle cell trait) are at approximately one-tenth of the risk of death from *P. falciparum* malaria compared with children who have *AA* genotype (homozygous for the normal gene)^10,24,39^. Lower levels of protection were reported in other studies^34,40,41^. Although some authors believe that the *SS* genotype (sickle cell disease) does not provide any protection against *P. falciparum*, for this simulation, a protection factor of 10 for both heterozygotes and homozygotes was assumed.

##### Effect of HbS on the fertility rate

Some researchers have suggested that women with *AS* genotype have a higher fertility than normal women^42,43^. Other studies, however, revealed no fertility difference between women with sickle cell trait and women with normal genotype in terms of completed family sizes, numbers of pregnancies, live births, or abortions^44^; by default, no increase in fertility of the heterozygotes was assumed in the simulation.

##### Overlap of parent and offspring populations

In most simulations so far presented, there was no overlap between the parent and offspring populations. In the current simulation, it was assumed by default that in each generation, 5% of parents may mate with members of offspring population.

### Simulation

#### Algorithm

Considering the parameter values discussed, the computer simulation program was developed based on the Monte Carlo method. The pseudocode of the program is shown in Table 3. In the first step of this simulation, for the very first generation, the elements of a 50-element one-dimensional array of integer numbers were corresponded to each person of a 50-person population (25 men and 25 women in reproductive age). The number assigned to each array element reflected the genotype of the corresponding person (0 = normal, 1 = heterozygote, 2 = homozygote). It was assumed that only one of the 50 persons (default value) was heterozygous for the gene — a mutant. In the second step, the gene frequency (*f*_*gene*_) and the frequencies of homozygous (*f*_*homo*_) and heterozygous (*f*_*hetero*_) individuals in the population at the zygotic level were calculated.

**Table 3:** Pseudocode of the simulation program.

1	Initialize the parent population with one heterozygous for the gene
	*loop* for each generation from 0 to 100
2	Calculate *f*_*gene*_, *f*_*hetero*_, *f*_*homo *_/* zygotic level */
3	Selection process: eliminate those who died of malaria and/or sickle cell disease
	*If* (overlap permitted)
4	Replace some of the population members with grandparents
5	Calculate *f*_*dead*_
6	Record the calculated frequencies
	*If* ((no homozygous *AND* no heterozygous) *OR* population size after selection < 2)
7	Record the generation at which “gene aborted” and *End*
8	Shuffle parent population array /* to provide a random mating */
	*loop* for each couple alive in parent population
9	Determine the number of children and their genotypes based on Mendelian inheritance
	*Endloop*
10	Calculate the size of the next parent population /* growth */
11	Reinitialize the new parent population based on the offspring population
	*Endloop*

A newborn in the hypothetical population ran a risk of *M*_*m*_ (15% by default) of dying of malaria before the reproductive age. Then, *M*_*m*_ × (1 *–* *f*_*hetero*_ *–* *f*_*homo*_) × 50 normal persons of the parent population would die of malaria before having offspring. The mortality probability from malaria in persons heterozygous for HbS, who are *P*_*hetero*_ (10 by default) times less likely to die of malaria, is *M*_*m*_*/P*_*hetero*_. Therefore, in the hypothetical population, *M*_*m*_ */* *P*_*hetero*_ × *f*_*hetero*_ × 50 heterozygotes would die of malaria. Assume that the mortality of those homozygous for HbS from the disease complications before reproductive age is designated as *M*_*ss*_ (85% by default). Furthermore, assume that homozygotes, like heterozygotes, are *P*_*homo*_ (10 by default) times less likely to die of malaria. Therefore, in the population, [*M*_*ss*_ + (1 – *M*_*ss*_) × *M*_*m*_ */* *P*_*homo*_)] × *f*_*homo*_ × 50 homozygotes would die of either malaria or sickle cell disease. In step 3 of the simulation, using these probabilities, those who would die of malaria and/or sickle cell disease were found for each genotype and eliminated from the population before mating.

In step 4 of the simulation, if the overlap between the parent and offspring populations was permitted, a fraction of the parent population (5% by default) was replaced by the members selected at random from the grandparent population. This was only done from the first generation onward as replacement of members of the zeroth generation (the very first parent population) with their parent’s population had no effect on the genetic structure of that population since all of them were presumably normal — the first mutation occurred in the zeroth generation.

In step 5, the frequency of dead people in the parent population was calculated. Some of the dead people after the selection process (step 3), might be replaced in step 4 by some people from the previous generation. All the calculated frequencies (steps 2 and 5) were then recorded for further analysis (step 6).

In step 7, it was determined if any gene carriers still remained in the parent population (after the selection and overlap processes). If no one homozygous or heterozygous for the gene remained or if the number of people in the parent population was less than two individuals, it was concluded that the gene was aborted and the generation at which it happened was recorded; else, the program proceeded to the next step.

In step 8, to produce the same chance for each survivor in the parent population to marry another, using a pseudo-random generator algorithm,^45^ the population array elements were shuffled.

In step 9, those of the parent population corresponding to even positions of the array (0, 2, 4, etc.) mated with those corresponding to their next element (positions 1, 3, 5, etc.) in the array to produce children according to their genotype and Mendelian inheritance; the genotype of each child was then determined. The number of children for each couple was determined at random from a lookup table determining the number of children and its probability for each couple depending on whether they were hunter-gatherer or farmer. It was assumed that couples had an average of four children (hunter-gatherer) for the first five generations; it was increased to five (farmer) thereafter.

In step 10, the size of the next parent population was calculated. In step 11, the members of the new parent population were selected at random from the offspring population. The whole process repeated from step 2 for 100 generations (~ 2500 years).

To eliminate the chaotic effects caused by inherent randomness of the Monte Carlo method^23^, the arithmetic mean of the values obtained from repeated consecutive executions of the program was taken as the final refined results. Simulations with initial 25 couples (default value) were repeated 10,000 times; with 2500 couples, 1000 times. To better understand the effect of each of the parameters studied, simulations with initial conditions other than the default values were also carried out (Tables 1 and 2).

## Data Availability

The pseudocode of the simulation program is presented in the manuscript; the source code developed in *C* programming language will be available on request from the corresponding author.
